# Who *cares* about migraine? Pathways and hurdles in the European region - access to care III

**DOI:** 10.1186/s10194-023-01652-8

**Published:** 2023-09-01

**Authors:** Gloria Vaghi, Roberto De Icco, Cristina Tassorelli, Peter J. Goadsby, Teófila Vicente-Herrero, Elena Ruiz de la Torre

**Affiliations:** 1 Department of Brain and Behavioral Sciences, University of Pavia, Pavia, Italy; 2 Headache Science and Neurorehabilitation Center, IRCCS Mondino Foundation, Pavia, Italy; 3 NIHR King’s Clinical Research Facility, King’s College London, London, UK; 4 ADEMA-SALUD University Institute of Health Sciences-IUNICS Illes Balears, Illes Balears, Spain; 5 EMHA, European Migraine and Headache Alliance, Brussels, Belgium

**Keywords:** Migraine, Headache, Burden, Disability, Pain, Quality of life, Chronic migraine, Access to care

## Abstract

**Background:**

Migraine is a highly prevalent primary headache disorder and a leading cause of disability. Difficulties in access to care during diagnostic and therapeutic journey contribute to the disease burden. Several target-specific drugs have reached the market in the past four years and have modified the treatment paradigm in migraine. The aim of this study is to provide an updated snapshot of the pathways and hurdles to care for migraine in different European countries by directly asking patients.

**Methods:**

In 2021 the European Migraine and Headache Alliance proposed a 39-item questionnaire that was administered online to an adult migraine population in European countries. Questions were focused on socio-demographic and migraine data, access to diagnosis and treatment, disease-related burden and the main channel for disease information.

**Results:**

A total of 3169 questionnaires were returned from 10 European countries. Responders were predominantly females, age range 25–59 years, with a migraine history longer than 10 years in 82% of cases, and with at least 8 headache days per month in 57% of cases. Respondents reported limitations in social, working and personal life during both the *ictal* and *interictal phase*. The activities mostly impaired during the attacks were driving (55%), cooking or eating (42%), taking care of family/childcare (40%) and getting medicines at the pharmacy (40%). The most frequently reported unmet need was the long delay between the first visit and migraine diagnosis: 34% of respondents had to see ≥ 4 specialists before being correctly diagnosed, and between the diagnosis and treatment prescription: > 5 years in 40% of cases. The most relevant needs in terms of quality of life were the desire for a lower migraine frequency, an effective treatment and a greater involvement in society.

**Conclusions:**

Data from the present survey point to the existence and persistence of multiple hurdles that result in significant limitations to access to care and to the patients’ social life. A close cooperation between decision makers, healthcare workers and patients is needed to overcome these barriers.

**Graphical Abstract:**

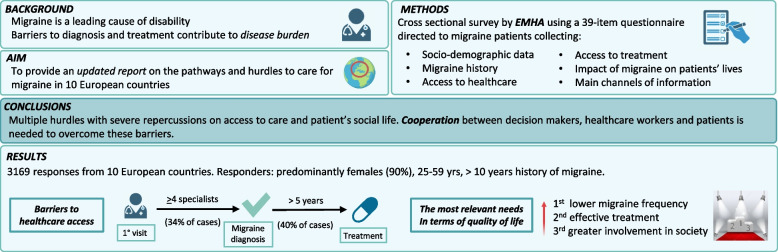

**Supplementary Information:**

The online version contains supplementary material available at 10.1186/s10194-023-01652-8.

## Background

Migraine is one of the most prevalent disorders worldwide, affecting a substantial number of patients, their families and the society at large [1, 2]. Migraine is a primary headache disorder specifically defined by recurring attacks with some combination of medium-severe intensity, unilateral, throbbing pain, generally worsened by routine physical activity. Attacks can last from 4 to 72 h when untreated and are often accompanied by photophobia, phonophobia, nausea, or vomiting [3].

Pain and associated symptoms during attacks may cause severe disability, known as *ictal burden* [4]. However, the migraine attack only represents the tip of the impact iceberg, as the *interictal burden* of disease takes the lion’s share in migraine disability*,* ranging from cognitive to psychological and emotional symptoms [5]. It should be noted that an ictal genesis of such non-painful symptoms, i.e. the premonitory phase, could not be completely ruled out, especially in high frequency episodic or chronic migraine. Indeed, previous studies demonstrated high prevalence and duration of premonitory or postdrome migraine components, that can last from several hours up to days with repercussions on headache free periods [6]. Migraine leads to restrictions in social, personal and working domains over many decades of life being characterized by high responsibilities [7–9]. It impacts interpersonal dynamics in social and family planning [10–12].

In this context, it is easy to understand why migraine is a pre-eminent cause of years lived with disability due to a disease among young women, and is second in the list of diseases causing disability overall, according to the 2019 Global Burden of Disease study [2].

Migraine is a treatable disorder and several acute and preventive therapeutic approaches are now available [13]. It is still largely underdiagnosed and poorly treated worldwide [14, 15], which has a negative impact on social life and workplace productivity [16, 17]. In addition, suboptimal medical approaches carry the risk of the development of chronic migraine [18], the most disabling phenotype across the migraine spectrum [3].

While the medical community is becoming more familiar with migraine-related *ictal burden*, the understanding and analysis of its *interictal* load is far less simple, less appreciated and often neglected by society and stakeholders. The fear of future attacks, associated with anticipatory anxiety and psychological repercussions, causes feelings of helplessness in patients. Their persistence also in crystal-clear days and their psychological aftermath carry the risk of overwhelming social and personal life [5, 19].

The patients’ voice has, over time, become more powerful, informing stakeholders about patient specific needs along with the hurdles they meet in obtaining appropriate care. The European Migraine and Headache Alliance (EMHA) is a lay non-profit organization that gathers 34 patient associations across Europe (www.emhalliance.org). Through channeling patient voices, it has contributed to increase awareness among stakeholders and society through multiple activities [20–22]. Collecting personal experiences about the hurdles faced on the path to diagnosis and treatment can be the first step to understand what should be recommended, or avoided, to improve migraine care.

Since 2019 the appearance on the European market of several target-specific migraine drugs has strengthened the therapeutic armamentarium for doctors, but it is not clear whether this has had a positive impact on patient needs. The aim of the present survey is to present an updated report on the pathways and hurdles to care for migraine in different European countries by means of a survey among a large population of migraine patients.

## Methods

This is a cross-sectional multinational survey initiated by EMHA. It is based on the administration of a questionnaire to patients with either episodic or chronic migraine. A draft version of the questionnaire was prepared by EMHA taking into consideration migraine-related issues that were deemed more relevant and critical for patients. The draft version was submitted to headache experts for content accuracy, relevance and novelty. The final version of the questionnaire was then made available in ten different languages and administered in European Countries with the support of local/transnational organizations for participants with migraine.

The questionnaire is composed of 4 different sections for a total of 39 items: 1) 7 items focused on socio-demographic data; 2) 6 items on migraine history; 3) 10 items on access to healthcare, 11 items on access to treatment and 3 on impact of migraine on the patient’s life; 4) 2 items on the main sources of information about the disease (supplementary material).

The survey was launched in March 2021 in most of countries. Data collection was completed in June 2021.

The questionnaire was sent through emails to members of patient organizations. The single, stringent inclusion criterion, was that the patient had been diagnosed with episodic or chronic migraine by a doctor. Participant consent was obtained prior to involvement in the survey and the questionnaires were collected by EMHA, which handled data confidentially and anonymized the respondents. For this reason, the survey was exempted from ethics committee review.

### Statistical analysis

Data analysis was conducted by a KPMG Life Sciences consulting team based in Spain. Categorical data are presented as the percentage of responses. Only countries that provided at least 100 questionnaires were included in the final statistical analysis. After removing the data from the countries that contributed less than 100 questionnaires each, the database was composed of 10 countries: Finland, France, Germany, Greece, Ireland, Italy, Latvia, Norway, Spain, UK. The data was checked for quality, which included an evaluation of response patterns and inconsistencies. Comparisons of different subset of participants from the 10 different countries were achieved with frequency distribution.

## Results

### Socio-demographic and migraine features of respondents

**Fig. 1:**
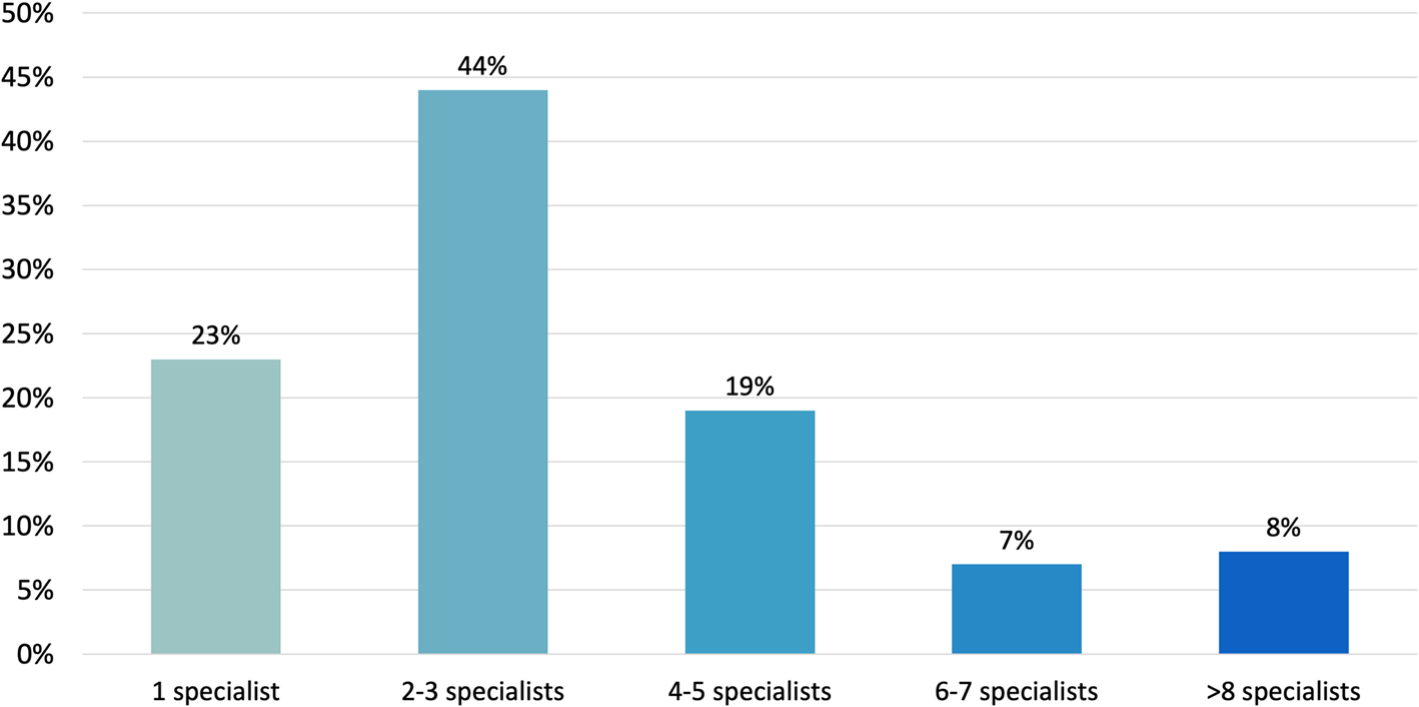
Number of specialists visited to get the final diagnosis. Total respondents N. 2519 (Question 3.1.2) (Percentages are approximated to the nearest unit)

A total of 3169 questionnaires were returned from the 10 European countries (Fig. 1 Suppl. Material). The average completion of the questionnaire items varied from a maximum of 99% for the first section to a minimum of 65% for the last one. Ninety percent of respondents were women, with an age range of 25–59 years. The majority of subjects lived in urban areas (67%); 48% of them were full-time employed, 18% part-time employed and 9% were self-employed. Declared family annual income according to the country of origin was below $US 40,000 (approximately equal to €35,000) in most of the cases, with few exceptions, notably Norway and Germany.

In one-third of respondents, migraine history was longer than 30 years, reflecting young age at disease onset. Nearly 50% of respondents suffered from chronic migraine, 18% from migraine without aura, 11% from migraine with aura and 15% from both migraine with and without aura. More than half of the subjects (57%) reported at least 8 monthly headache days in the previous 3 months. Complete socio-demographic and migraine features are reported in Table 1.

**Table 1:** Socio-demographic features of respondents (data expressed as %)

**Sex**	
Female	90
**Age (years)**	
< 18	0
18–24	4
25–44	46
45–59	40
60–70	9
> 70	1
**Living environment**	
Urban	67
Rural	33
**Employment status**	
Employed full-time	48
Employed part-time	18
Self-employed	9
Unemployed	15
Retired	10
**Years lived with migraine**	
< 5	5
5–9	13
10–19	25
20–29	25
> 30	32
**Monthly headache days**	
< 4	16
4–7	27
8–14	26
> 14	18
Daily	13

### Healthcare and treatment access

The large majority of respondents saw at least two specialists before receiving a migraine diagnosis (67%) and 34% had to see four or more specialists to achieve the final diagnosis (Fig. 1). Finland, Norway and Ireland had the lowest number of specialists seen to get a correct diagnosis. In these countries, migraine diagnosis was achieved in a higher percentage of cases by general practitioners, namely in 50%, 35% and 31% of cases, respectively. In contrast, patients from Italy, Greece and Latvia reported a higher percentage of specialized consultations before reaching a final diagnosis: more than 4 specialists in 54%, 35% and 46% of cases, respectively. In these countries, the diagnosis was usually made by neurologists.

**Fig. 2:**
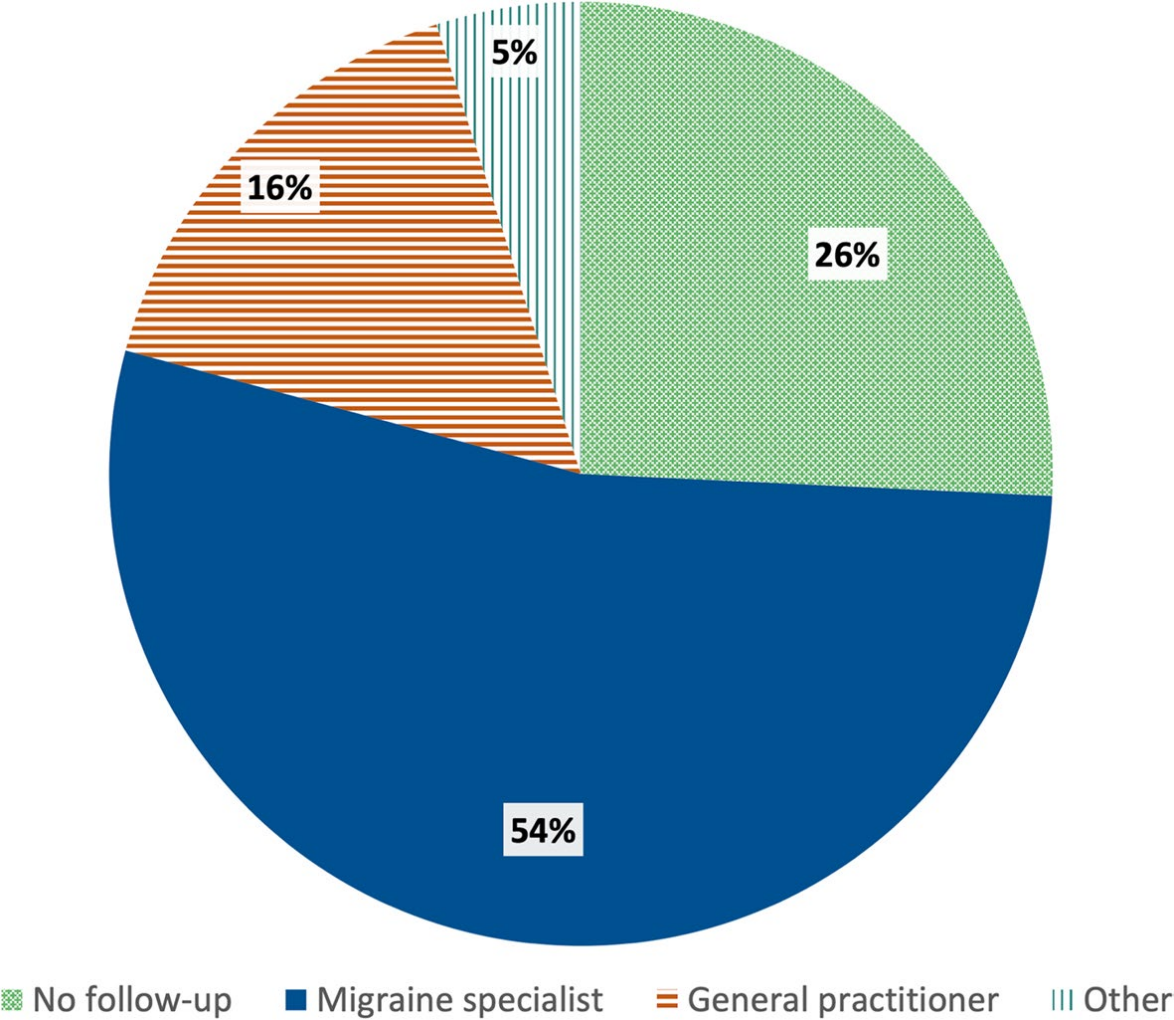
Follow-up of migraine patients. Total respondents N. 2509 (Question 3.1.4) (Percentages are approximated to the nearest unit)

**Fig. 3:**
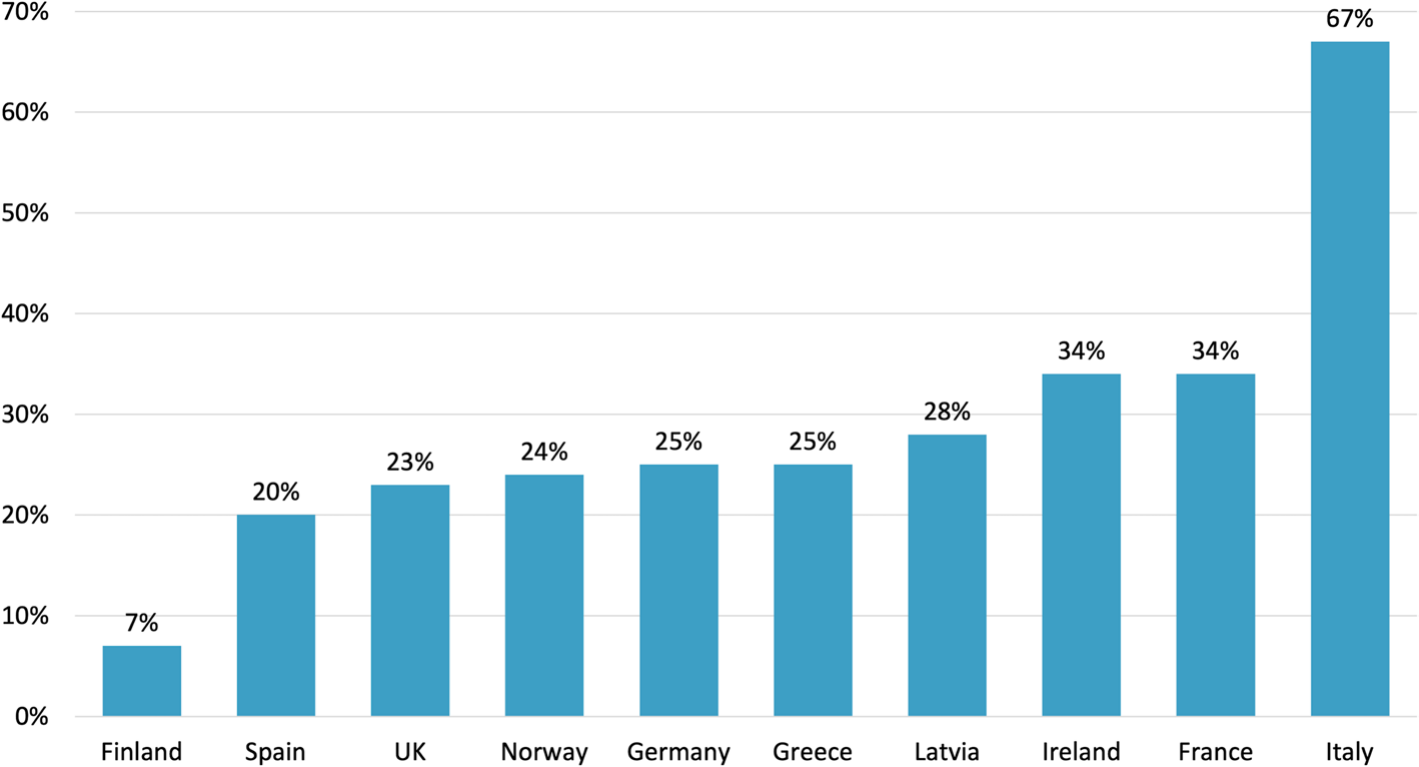
Percentage of patients who underwent follow-up in a specialized center according to country of origin. Total respondents N. 2514 (Questions 3.1.7)

Neurologists performed follow-up in 54% of cases (Fig. 2). Once diagnosed, the majority of patients were followed by neurologists in many countries, with the exception of UK, Finland, Latvia and Ireland, where the percentage of patients followed by other healthcare professionals, or not being followed by any healthcare professional, was larger than the percentage of patients who were followed by neurologists. Different percentages were also evident among patients followed-up in specialized headache centers (Fig. 3).

Only 37% of patients were actually prescribed medications specifically for migraine within 1 year from the diagnosis, while it took more than 5 years in 40% of cases. Acute non-specific medications were the first treatment prescribed (in 46% of cases), followed by triptans, which were prescribed in 78% of respondents. Oral preventive drugs were prescribed as a third-line choice. Advanced therapeutic strategies, such as monoclonal antibodies (MABs) targeting calcitonin gene-related peptide (CGRP) and onabotulinum toxin A (BoNTA), were prescribed only after several other therapeutic options (up to 6 for MABs). Eleven % of respondents were receiving BoNTA treatment, while 24% were being treated with MABs. The major barriers to migraine specific treatments (MABs and BoNTA) in recent years were COVID-19 pandemic, lack of treatment subsidization by local health system and social stigma. Among those respondents who were not on MABs treatment, nearly 60% knew about the existence of these migraine-specific drugs. They reported that these treatments were not prescribed to them because the doctors did not mention the option during the visit, or they did not meet the eligibility criteria or, again, treatment cost was not covered by their national health system (Table 2). Of note, 82% of subjects who were undergoing treatment with MABs were satisfied with their effectiveness.

**Table 2:** Main barriers to access to monoclonal antibodies targeting CGRP (MABs) according to responses from subjects who were not on MABs treatment (%). Total respondents N. 1684 (Question 3.2.3 b)

Reason	(%)
Not mentioned by the doctors	26
Not covered by the health system	25
Not meeting eligibility criteria	25
Not available in the home country	10
Not known by doctors	6
Not indicated	8

The length of the diagnostic and therapeutic process has financial repercussions as more than half of respondents (52%) declared an impact of treatments and visits on their income.

Social media and the web were the main sources of information about diagnosis and treatments.

### The most important needs

Upon direct request, respondents classified having a lower migraine frequency (29%), an effective treatment (23%) and a greater involvement in society (22%) as the most important needs in terms of quality of life. These were followed by the desire for a lower headache severity (14%), a more productive life (13%) and an improvement of family life (9%).

During the *ictal phase,* subjects indicated the need for an external help in several social and working activities. Driving was the most frequently and consistently impaired activity during attacks, imposing the need to ask for support by others in 55% of cases, immediately followed by food consumption (42%), taking care of family (40%) and getting medicines (40%). Working was also affected with more than 30% of subjects asking for replacement at work during a migraine attack. Making business calls and drug consumption were affected in around 10% of cases.

Interestingly, 10% of respondents also declared to need help in driving, food consumption and taking care of family during the *interictal phase*.

## Discussion

The present survey provides a comprehensive set of data from a large number of migraine patients across different European countries. It provides first-hand information on migraine diagnostic and therapeutic processes, their hurdles and the most relevant needs in terms of quality of life.

Our cohort was mostly composed of women in their second-to-fourth decades of life, reflecting epidemiologic features of migraine [19]. Our sample seems adequate for the objectives of the study, as females from 15 to 49 years old were recognized as the most disabled in terms of years lived with disability (YLDs), a quantification of time lost because of migraine [23].

Our analysis focused on migraine disability, a key indicator consisting of *ictal* and *interictal burden,* known to correlate inversely with the quality of migraine care [24].

In our cohort, migraine *ictal burden* was reported by almost half of respondents, described as limitations in driving, food consuming and taking care of family. Notably, the same daily activities were affected in 10% of respondents even outside attacks, denoting the *interictal burden*.

Both *ictal* and *interictal burden* may lead to anticipatory anxiety, outlining limitations on personal life, productivity and social planning, as well as negatively affecting social relationship and the whole family environment [5, 7–12]. A recent survey conducted by the Greek Society of Migraine and Headache Patients underscored the relevant burden of migraine [12]. Nearly 70% of respondents reported relevant limitations in emotional, social, working and financial domains. This finding is in agreement with a previous observational study conducted on a large sample of the Greek population interviewed by means of a computer-assisted telephone interview, where 64.3% of subjects with migraine reported that headaches prevented them from responding to their social or family obligations at least 1 day per month [9]. Our present data complement these previous findings reported by bringing into the picture the perspective of patients from multiple European Countries and by listing the daily activities that were actually impaired by migraine, also interictally.

In our survey, the severity of the *interictal burden* may be underestimated due to the closed-questions method adopted, which was necessary to manage free-text information in this large survey. Because of its multifaceted and psychological nature, *interictal burden* may be better captured through open questions or validated scales [4] in ad hoc targeted studies. Notably, a prodromal or postdromal origin of such non-painful symptoms could not be ruled out. Targeted questions about the temporal relationship between non-painful symptoms and headache attacks could be relevant to discern between *interictal* or migraine attack phases. Migraine *ictal* and *interictal* burden is important when considering that patients indicated being “more included into the society” as the third most relevant need in terms of quality of life. This response also sheds light on the social and work stigma, namely the challenge to communicate to other people, including co-workers, one’s own challenges and to reach their acceptance [5, 9, 12, 25].

A reduced headache frequency and an effective treatment emerge as the most important needs related to quality of life. High frequency migraine and lack of specific acute and preventive treatments contribute to reduced productivity during attacks and higher anticipatory anxiety between them. Both conditions are strictly connected to access to care and consequent proper diagnosis and follow-up. Indeed, difficulties in getting a diagnosis and a proper management inevitably determine an increased rate of absenteeism with socio-economic impact due to healthcare visits and sick leaves [26]. Of note, more than one-third of our respondents declared a process longer than 5 years to get an acute or preventive treatment.

Lack of appropriate follow-up is another relevant unmet need. Difficulties in this area range from complete lack of follow-up to low referral to specialized headache centers, reflecting data from previous surveys conducted in 2008 and 2013 [27]. Notably, though migraine is climbing the ranks of years lived with disability, the access to care gap does not seem to be reducing in parallel. This seems even more relevant when considering the present availability of novel target-specific and effective treatments, like the MABs targeting CGRP, gepants and ditans. The data from our cohort show that MABs targeting CGRP were prescribed only after several other treatment options. Actually, due to their high cost, prescription is usually entrusted to specialized headache centers and, when subsidized by the national health systems, strict criteria need to be satisfied. Though country-specific, prescription criteria mainly focus on the number of monthly migraine days, without taking into consideration the degree of disability associated with them. According to the group of respondents who had never been prescribed MABs, the main reasons were physicians not mentioning the option, lack of subsidization by the healthcare system and the fact that they did not meet eligibility criteria. It is intuitive that the two latter reasons are highly likely to have an impact on the access to drugs with proved efficacy in episodic and chronic migraine, and are more related to criteria of economic prioritization rather than to scientific evidence. A precise analysis of access to care in relation to healthcare coverage is beyond the scope of this paper, but it is definitely an issue worth of attention.

Remarkably, MABs users reported a high percentage of treatment satisfaction (82%). A lower percentage of satisfaction with MABs (64%) was reported in the before-mentioned Greek survey, potentially related to the high disparity on MABs users among the two studies (24% vs 3% of the total respondents) [12].

“COVID-19”, “budget constraints” and “social stigma from policy makers” were acknowledged as the most relevant barriers to get access to specific treatments. Among organizational, financial or social barriers [28], the COVID-19 pandemic increased the challenge in managing chronic conditions [29]. These findings are in agreement with Dermitzakis et al., who reported the impact of pandemics on the attendance of regular follow-ups [12].

Interestingly, an analysis of our data based on the country of origin allowed identification of discrepancies in the management of migraine. Countries where migraine was diagnosed by general practitioners were the ones where less specialist visits were necessary. This was particularly true for Norway, Finland and Ireland, the areas where less patients are referred to a specialized center. This may be due to a limited number of specialists in those countries, with the ensuing increase in general practitioners commitment in headache diagnosis and treatment [30]. However, in a new environment where target-specific migraine drugs are prescribed by specialists, the lack of specialized centers could result in reduced therapeutic options.

At the opposite pole stands Italy with more than two-thirds of patients followed in specialized centers. This country follows the headache care model subdivided in first, second and third level centers, according to the European Headache Federation recommendations, with second and third level centers offering advanced migraine care [31]. Theoretically, general practitioners are entrusted with the crucial role of *gate-keepers* in balancing demand and need (patients seeking specialized health care and patients who would benefit from it) and correctly triaging patients to the right service [32]. This does not always happen in the real world, due to several reasons that are beyond the focus of this study. In the absence of the essential role of *gate-keepers,* patients directly access second and third levels of care, with the consequence of growing costs, longer waiting times, reduced efficiency and the risk of denying specialist access to those in need. It is evident that, in this situation, general practitioners should be empowered with appropriate skills, active commitment and a direct dialog to subsequent levels of care. This would guarantee specialized medical approach to those in need and a fast access to novel target treatments.

Source of information represents another important stage in access to care. As social media and internet represent the main sources of information according to our survey, careful curation of information seems essential for a correct approach to migraine. As most patients’ organizations use the internet as a mean of communication, they should act as a *compass* in providing correct and clear information to help patients in their diagnostic and therapeutic journey. A virtuous collaboration between general practitioners, specialized centers and patient organizations would be advisable for proper management of patients’ needs.

### Limitations

Data collection based on a self-report survey carries an intrinsic limitation in terms of accuracy. For example, there is a possible discrepancy between the self-declared diagnoses and ICHD-3 criteria [3]. Nonetheless, a physician-administered questionnaire, though more precise, would have excluded respondents without actual follow-up at the time of the survey.

On the other hand, source of data collection, only among patients’ organization, could be linked to a *selection bias*. The members of patient organizations are usually people moderately to highly disabled by migraine. Thus, this survey is mainly centered on a particularly disabled population actively seeking medical attention, with a possible underestimation of access to care barriers.

Moreover, generalization of relevant needs and expectations could vary upon culture, past history and resources available in the country of origin, thus caution should be used in results interpretation and generalization.

## Conclusions

The present survey provides insights from a large cohort of migraine patients from several European Countries. It corroborates the existence of different hurdles analyzed through patients’ perspectives, all determining severe repercussions on access to care and patients’ social life.

Our results suggest the need of a continuous and multi-stakeholder action in which decision makers, healthcare providers, healthcare professionals, patients’ organizations and representatives join forces to overcome these barriers.

### Supplementary Information

**Additional file 1:** Complete questionnaire & Figure 1 Suppl. Material.

## Data Availability

The datasets used and/or analyzed during the current study are available from the corresponding author on reasonable request.
