# Supplementary material for: Who cares about migraine? Pathways and hurdles in the European region - access to care III
Source: J Headache Pain. 2023 Sep 1;24(1):120. doi: 10.1186/s10194-023-01652-8 (PMC10472594; doi:10.1186/s10194-023-01652-8)
Supplement: Supplementary file 1 — Additional file 1: Complete questionnaire & Figure 1 Suppl. Material. [file 10194_2023_1652_MOESM1_ESM.docx]

**Supplementary material**

**Figure 1S**– Countries of origin of respondents. Total respondents N. 3169 (Question 1.1)

**Access to Care III Survey Questionnaire**

**1. SECTION I: ABOUT YOURSELF**

1.1. Country of residence

- Spain
- Italy
- Germany
- Portugal
- Ireland
- UK
- Czech Republic
- Norway
- France
- Greece
- Austria
- Finland
- Latvia
- Other (please specify):___________

1.2. Gender

- Man
- Woman
- Other

1.3. Age

- 18-24
- 25-44
- 45-59
- 60-70
- Over 70

1.4. Area of residence

- Urban (City)
- Rural (Countryside)

1.5. Can you please specify approximatively the level of your FAMILY annual income? Please answer the survey without taking into account the current COVID19 constraints. Your answers should reflect your experience as a patient out of COVID19 pandemic situation.

- No income
- Less than 40,000€ (or equivalent in other currencies)
- Between 40,000€ to 60,000€ (or equivalent in other currencies)
- Between 60,000€ and 80,000 €/year (or equivalent in other currencies)
- More than 80,000 €/year (or equivalent in other currencies)
- I prefer not to say

1.6. What is your employment status?

- Self-employed
- Employed part-time in an organisation
- Employed full -time in an organisation
- Unemployed
- Retired

1.7. What is your general health coverage?

- Private
- Public
- Both

**2. SECTION II: ABOUT YOUR MIGRAINE**

2.1. Please specify your type of Migraine

- With Aura
- Without Aura
- Both types
- Chronic migraine
- Not sure
- Other (please specify): _________________________

2.2. For how many years have you been suffering from Migraine?

- Less than 5 years
- Between 5 years and 10 years
- Between 10 years and 20 years
- Between 20 years and 30 years
- More than 30 years

2.3. Over the last 3 months, how many days of migraine do you have per month on average?

- Less than 4/ month
- Between 4 and 7/month
- Between 8 and 14 /month
- More than 14 / month
- Daily pain

2.4. On the days that you have an attack, do you need someone to help you with one or more of the activities listed below? Select “Yes” – “No” – “Sometimes”

- Administration of the treatment for migraine
- Cooking or Eating
- Taking care of Family/Childcare
- Getting medicines at the Pharmacy
- Driving
- Calling Employer
- Coworkers to help/replace your workplace

2.5. On the days that you DO NOT have an attack, do you need someone to help you with the activities listed below? Select “Yes” – “No” – “Sometimes”

- Administration of the treatment for migraine
- Cooking or Eating
- Taking care of Family/Childcare
- Getting medicines at the Pharmacy
- Driving
- Calling Employer
- Coworkers to help/replace your workplace

2.6. What is the most important issue that you want to address in terms of quality of life due to your migraine? Please rank from 1 to 6. (Where 1 is the less important and 6 the most important) (*)

- To have less migraine days per month
- To have less severe migraine attacks
- To have an effective treatment
- To improve my family life
- To be more productive at work
- To be more included into the society

**3. SECTION III: ABOUT YOUR ACCESS TO CARE**

3.1. Access to Healthcare Professionals:

3.1.1. What has been the first specialist you visited due to your migraine?

- General Practitioner
- Internist (Internal Medicine)
- Neurologist
- Migraine specialist
- Other (please specify)

3.1.2. How many specialists have you visited to get the final diagnosis?

- 1
- 2 or 3
- 4 or 5
- 6 or 7
- More than 8

3.1.3. Which specialist made your first migraine diagnosis?

- General Practitioner
- Internist (Internal Medicine)
- Neurologist
- Migraine specialist
- Other (please specify):________________

3.1.4. Are you regularly followed by a Healthcare Professional because of your migraine?

- No, I am not followed by any healthcare professional
- I am followed by a Nurse
- I am followed by a G.P. or a family Doctor
- I am followed by a Headache Specialist (neurologist)
- I am followed by other type of Specialist, please specify (open answer):___

3.1.5. If you are followed by a physician because of your migraine, please specify the healthcare setting:

- Public health care system
- Private health care system

3.1.6.Who pays for your physician visit in relation to your migraine?

- The public health care system
- I pay out of my pocket
- I have a private insurance
- A combination of the above

3.1.7.Do you go to a specialised migraine center?

- Yes
- No

3.1.8. If Yes to previous question: How easy was it to get a visit at the migraine center? (scale from 1 to 5: 1 = Very difficult and 5: Very easy)

3.1.9. If yes to previous question: How have you found this migraine center?

- Social media (e.g. Facebook, Twitter, Instagram)
- Internet browser (e.g. Google, Bing, etc.)
- People or friends
- Patient associations
- My doctor
- A nurse
- The pharmacist
- Pharmaceutical Companies
- Migraine Apps

3.1.10. If you need to contact a Health Care Provider when suffering an attack, do you have immediate access to ... Select “Always” “Very often” “Sometimes” “Rarely” “Nearly never” “I don’t know”

- To your G.P.
- To a Specialist like Neurologist
- To a physical therapist, psychologists, or other therapists
- To Hospitals Services
- To a nurse

3.2. Access to Treatments:

3.2.1. Do you get a treatment for your Migraine?

- YES
- NO

3.2.2. If YES:

a) How much time since your diagnosis of migraine until you received treatment for your migraine?

- 1year
- 2-3years
- 3-5years
- More than 5 years

b) Which treatments have you taken since your diagnosis (check all that apply in chronological order – 1 to 7)?

- Symptomatic over the counter medications (painkillers or general analgesics) when I have a migraine attack.
- Specific prescription medications for acute migraine attack (e.g. TRIPTANS)
- Preventive treatment (a drug taken regularly to reduce the number of migraine attacks) (e.g. Topiramate)
- Botulinumtoxin (Botox) preventive treatment
- New Anti-CGRP preventive treatments (these are new injectable biological drugs taken regularly to reduce the number of migraine attacks)
- Both symptomatic and preventive treatments
- Other complementary therapies (Acupuncture, physical therapy, herbal products, homeopathy, etc.).

c) What is your current treatment? (check all that apply)

- Symptomatic over the counter medications (pain killers or general analgesics) when I have a migraine attack.
- Specific prescription medications for acute migraine attack(e.g. TRIPTANS)
- Oral preventive treatment (a drug taken regularly to reduce the number of migraine attacks) (e.g. Topiramate)
- Botulinumtoxin (Botox) preventive treatment
- New Anti-CGRP preventive treatments (these are new injectable biological drugs like drugs taken regularly to reduce the number of migraine attacks)
- Both symptomatic and preventive treatments
- Other complementary therapies (Acupuncture, physical therapy, herbal products, homeopathy, etc.).

3.2.3. If the respondent DIDN’T select the Anti-CGRP option they should answer the following questions:

a) Have you heard about new Anti-CGRP preventive treatments (these are new injectable biological taken regularly to reduce the number of migraine attacks)?

- Yes
- No

b) If your answer to previous question was ‘yes’, why don’t you have access to new CGRP specific preventive treatments? Please tick the situation that best applies you

- Because they are not covered by my health system (national health system or private insurance) in my country our healthcare system has put a cap on access to Anti-CGRP treatments
- Because my doctor doesn’t know about it
- Because according to my doctor I don’t need it
- Because my doctor didn’t mention it
- Because they are not available in my country
- Because I am not eligible for this treatment yet

If you selected the Anti-CGRP option in previous question, please answer the following question:

c) Who is paying for your treatment?

- I pay for Anti-CGRP preventive treatment out of my pocket
- The cost of Anti-CGRP preventive treatment is fully covered by Social Security
- The cost of Anti-CGRP preventive treatment is fully covered by Private Insurance
- The cost of Anti-CGRP preventive treatment is fully covered by a combination of both Social Security and Private Insurance
- The cost Anti-CGRP preventive treatment is partially covered.

Please specify by whom (Social security / Private Insurance / combination of both).

d) Are you satisfied with the results obtained from the new preventive CGRP treatment?

- I am not satisfied with the results of the treatments
- I have managed to improve but I still suffer limitations in my daily life
- I have improved a lot in my limitations of daily life
- I am very satisfied with the results of the treatments

3.2.4. From 1 – 5 can you rank what are the biggest (5) and the lowest (1) difficulties to get access to specific migraine treatments?

- My doctor does not give enough importance to migraine (stigma within the healthcare community)
- National healthcare system does not cover treatments for migraine
- Private insurance does not cover treatments for migraine
- Product is not available in the hospital formulary or in the migraine center
- Stigma from policy-makers who do not see migraine as a life-threatening disease and therefore do not think that prevent treatments should be reimbursed
- Budget constraints in my country which put limitations or caps to migraine treatments
- COVID19

3.2.5. Did you face the same difficulties for all treatments for migraine? If no, please give a score from 1 to 5 to each treatment you have received based on the difficulties to get access to it (use 5 for biggest difficulties and 1 for lowest difficulties)

- Symptomatic over the counter medications (painkillers or general analgesics) when I have a migraine attack.
- Specific prescription medications for acute migraine attack (e.g. TRIPTANS)
- Preventive treatment (a drug taken regularly to reduce the number ofmigraine attacks) (e.g. Topiramate)
- Botulinumtoxin (Botox) preventive treatment
- New Anti-CGRP preventive treatments (these are new injectable biological drugs taken regularly to reduce the number of migraine attacks)
- Both symptomatic and preventive treatments
- Other complementary therapies (Acupuncture, physical therapy, herbal products, homeopathy, etc.).

3.2.6. To help you managing better your migraine, please indicate from 1 to 5 which proposals could improve your situation (1 = doesn’t improve much; 5 = improve a lot)

- Increased knowledge and training for Healthcare Professionals about Migraine
- Increased awareness of the disease and its limitations to general population
- Increased awareness of the disease and its limitations Politicians / Payers
- Increased awareness of the disease and its limitations Employers
- Increased availability of specialized migraine centers
- Increased access to preventive treatments
- Accelerated path to get an effective treatment
- Other (please specify) ________________________

3.3. Impact of Patient’s life:

3.3.1. How much do you spend each month, out of your pocket as an average to treat Migraine? (private doctors, medicines, complementary treatments such as physiotherapy or mindfulness...etc.)

- Nothing
- Below 50 €
- Between 50 € and 100€
- Between 100 € and 200 €
- Between 200 and 300 €
- More than 300 €

3.3.2. Have you switched treatment or asked the physicians for other therapeutic alternatives because treatment cost is too expensive?

- Yes
- No

3.3.3. Does the cost of treatments for your migraines have a relevant impact on your finances?

- Yes
- No

**4. SECTION IV: SOURCES OF INFORMATION AND STIGMA (*)**

4.1. What is your main source of information about migraines?

- I do not have any information
- Social media (e.g. Facebook, Twitter, Instagram)
- Internet browser (e.g. Google, Bing, etc.)
- Other people or friends
- Patient association
- Doctor
- Nurse
- Pharmacist
- Pharmaceutical Companies
- Migraine apps
- Other (please specify)

4.2 What is your main source of information for migraine treatments?

- I do not have any information
- Social media (e.g. Facebook, Twitter, Instagram)
- Internet browser (e.g. Google, Bing, etc.)
- Other people or friends
- Patient association
- Doctor
- Nurse
- Pharmacist
- Pharmaceutical Companies
- Migraine apps
- Other (please specify)
